# Endotracheal Surfactant Combined With Budesonide for Neonatal ARDS

**DOI:** 10.3389/fped.2020.00210

**Published:** 2020-05-05

**Authors:** Burak Deliloglu, Funda Tuzun, Merve Meryem Cengiz, Hasan Ozkan, Nuray Duman

**Affiliations:** Division of Neonatology, Department of Pediatrics, Dokuz Eylul University Faculty of Medicine, Izmir, Turkey

**Keywords:** neonatal ARDS, Montreux criteria, surfactant plus budesonid, ELGAN Extremely low gestational age newborn, surfactant

## Abstract

Acute respiratory distress syndrome (ARDS) is a clinical condition characterized by acute diffuse inflammatory lung injury and severe hypoxemia. In 2017, the Montreux Consensus defined diagnostic criteria for ARDS in the neonatal period. The management of ARDS includes strict adherence to lung-protective ventilation strategies and therapeutic agents to improve gas exchange. We report two similar cases of premature infants with gestational ages of 23 and 24 weeks diagnosed with neonatal ARDS according to the Montreux definition. These patients developed acute worsening of oxygenation on the 30th and 28th day of life, respectively, while they were ventilated on volume-guarantee assist/control mode. Chest X-rays revealed bilateral diffuse opacity, there were no cardiogenic origins for pulmonary edema, and their oxygenation indexes were >8. Both cases fulfilled the neonatal ARDS criteria and the patients' clinical conditions were associated with late onset neonatal sepsis. After lung recruitment maneuver, the infants began HFO volume-guarantee ventilation and received surfactant treatment. Since they showed a poor short-term response, intratracheal surfactant of 100 mg/kg plus budesonide of 0.25 mg/kg were administered and their oxygenation indexes were reduced stepwise. Both patients survived and were discharged home with spontaneous breathing of room air. Neonatal ARDS is generally an underdiagnosed condition associated with sepsis, pneumonia, and meconium aspiration. Impaired surfactant activity and reduced lung compliance play important roles in its pathophysiology. To our knowledge, this is the first case report indicating the possible therapeutic role of budesonide plus surfactant in ARDS treatment. Since ARDS is an entity not recognized in newborns, we want to emphasize neonatal ARDS diagnosis and underline that the combination of budesonide and surfactant may be a novel therapeutic option in the treatment of ARDS.

## Introduction

Acute respiratory distress syndrome (ARDS) is a clinical condition characterized by acute diffuse inflammatory lung injury and severe hypoxemia. Neonatal ARDS is a novel diagnosis for newborns. De Luca et al. defined the Montreux Consensus diagnostic criteria for ARDS in the neonatal period for the first time (1). Although specific treatments do not exist other than the treatment of the underlying disease, the management of ARDS includes strict adherence to lung-protective ventilation strategies and therapeutic agents to improve gas exchange. Exogenous surfactant treatment has been considered to be beneficial in pediatric cases because of the importance of the impaired surfactant activation during the course of ARDS (2). The increased inflammatory status of the lungs is a central reference point in the pathophysiology of ARDS. Considering its potent local pulmonary anti-inflammatory effect, budesonide may be an effective treatment option for ARDS (3).

In this report, we present two cases of neonatal ARDS in newborns of extremely low gestational age treated successfully with endotracheal surfactant plus budesonide. Written informed consent was obtained from the parents of both patients for the publication of these case reports.

## Case Reports

### Case 1

**Figure 1:**
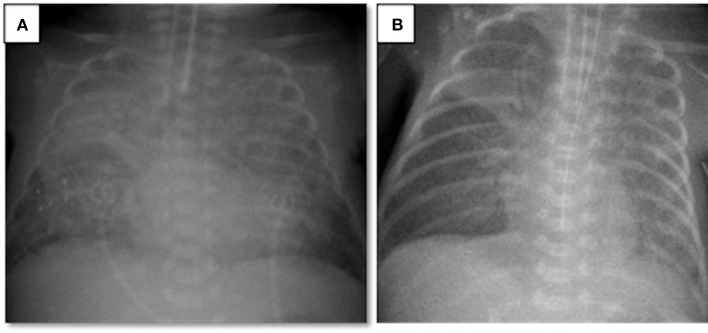
Chest radiograms of Case 1: **(A)** At the time of ARDS diagnosis, an X-ray revealed bilateral diffuse lung opacities; **(B)** after two doses of surfactant plus budesonide treatment, a chest X-ray revealed better aeration.

A 497-g triplet preterm female infant was born vaginally at the 23rd gestational week. The patient was intubated in the delivery room and received surfactant. Her Apgar scores at 1 and 5 min were 3 and 5, respectively. During the acute phase of respiratory distress syndrome, the infant was given two doses of surfactant and required high-frequency oscillatory ventilation plus volume guarantee (HFOV-VG) as a rescue therapy. Between the 3rd and the 30th day of life (DOL), the infant remained on ventilator therapy despite two unsuccessful extubation attempts, which failed due to patent ductus arteriosus and late-onset neonatal sepsis. Although the patient was clinically stable for at least a week and ventilated on conventional assist/control mode with volume guarantee at FiO_2_ of 0.3, on the 30th DOL the patient's oxygen requirement doubled and her FiO_2_ levels reached 0.6 within 12 h. A chest X-ray revealed bilateral diffuse lung opacities (Figure 1A), while blood gas analyses revealed respiratory acidosis with an oxygenation index of 12. To clarify this acute worsening, a full sepsis workup was completed including routine blood tests (complete blood count, peripheral blood smear, C-reactive protein), cultures from body fluids, tracheal aspirate culture, and respiratory viral PCR tests for influenza, parainfluenza, respiratory syncytial virus, metapneumovirus, coronavirus, rhinovirus, enterovirus, parechovirus, adenovirus, and bocavirus. Laboratory tests revealed an elevated C-reactive protein level (68 mg/L) and an elevated immature/total leukocyte ratio (0.41). The tracheal sample culture was sterile and viral PCR test results were negative. Echocardiography was performed, which revealed patent foramen ovale without any cardiogenic deterioration. The patient was diagnosed with neonatal ARDS on the basis of the acute worsening of her respiratory condition, diffuse opacities in the lungs as shown by radiography, the absence of pulmonary edema of cardiac origin, and an oxygenation index above four. After neonatal ARDS diagnosis, the patient underwent a lung recruitment maneuver under HFOV-VG to provide optimal lung volumes. To improve oxygenation, endotracheal surfactant was administered, which briefly decreased the oxygen requirement within hours; however, the FiO_2_ level again reached 0.6. In the presence of short-lasting clinical response to surfactant treatment, alternative treatment options were considered due to rapid surfactant inactivation. Considering ARDS, a therapy combining surfactant of 100 mg/kg (beractant, Survanta Bovine, Ross/Abbott Laboratories, Columbus, OH, USA) and budesonide of 0.25 mg/kg (Pulmicort nebulizing suspension, Astra Zeneca, London, UK) was administered via endotracheal route twice in an 8 h interval (4). The oxygenation index dropped to three after 12 h, the FiO_2_ level decreased to 0.35, and chest X-rays revealed better aeration (Figure 1B). In this case, with the support of laboratory findings, the ARDS etiology was determined to be clinical late-onset neonatal sepsis, although all cultures remained sterile. After the treatment, the patient's ventilator settings improved, and the patient could be weaned from the ventilator to non-invasive support on the 48th DOL. The patient was discharged to home on the 95th DOL without oxygen supplementation. The other two infants of this set of triplets died in the first week of life due to severe respiratory insufficiency.

### Case 2

**Figure 2:**
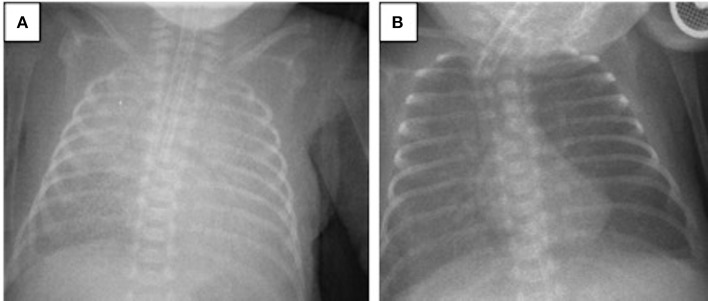
Chest radiograms of Case 2: **(A)** At the time of ARDS diagnosis, an X-ray revealed bilateral irregular diffuse opacity; **(B)** improvement in lung aeration was seen after one dose of surfactant plus budesonide treatment.

A 694-g twin preterm male infant was born via cesarean section at the 24th gestational week. The patient was intubated in the delivery room and received surfactant. His Apgar scores at 1 and 5 min were 4 and 6, respectively. During the first 2 weeks of life, the patient required HFOV-VG as a rescue therapy. Despite late systemic steroid treatment for weaning from the ventilator, he did not tolerate extubation. On the 28th DOL, the patient developed acute worsening of oxygenation with a FiO_2_ need of 0.75 under assist/control volume-guarantee ventilation. Blood gas analysis revealed respiratory acidosis and the patient's oxygenation index was 10. Chest X-rays revealed bilateral irregular diffuse opacity (Figure 2A), and there was no cardiogenic origin for pulmonary edema, as confirmed by echocardiography. The patient met the criteria for neonatal ARDS, including acute onset hypoxemic respiratory failure, diffuse bilateral lung opacification, absence of pulmonary edema due to cardiogenic disease, and an oxygenation index exceeding four. Tracheal aspirate samples obtained for bacterial culture and viral PCR tests were negative. His diagnosis of ARDS was also associated with a coexisting blood culture that proved late-onset neonatal sepsis, with *Staphylococcus epidermidis* as the pathogen. After a lung recruitment maneuver, the patient was ventilated on HFOV-VG mode and received a dose of 100 mg/kg surfactant (Poractant alfa, Curosurf, Chiesi Pharmaceuticals, Parma, Italy) plus 0.25 mg/kg budesonide (Pulmicort nebulizing suspension, Astra Zeneca, London, UK) endotracheally. As a result, his oxygenation index reduced stepwise to 2.6, the FiO_2_ requirement decreased to 0.4, and chest X-rays revealed better aeration (Figure 2B). Extubation was successful on the 43rd DOL and the patient was discharged to home without respiratory support on the 76th DOL.

## Discussion

The diagnosis of neonatal ARDS is based on the Montreux criteria, which implicate the timeframe, oxygenation index, origin of pulmonary edema, and lung imaging. To fulfill the criteria, a patient's clinical status has to be acute onset (i.e., within a week), with an oxygenation index >4 and lung imaging showing diffuse bilateral opacification with no cardiogenic origins for pulmonary edema, as confirmed by echocardiography (1). ARDS became slightly more recognized by neonatologists after the publication of the Montreux criteria, but it still remains an underdiagnosed condition in neonatal intensive care units (5). Both of our patients, as newborns of extremely low gestational age, developed neonatal ARDS during their NICU stay.

The incidence of ARDS is 2.9–9.5 per 100,000 and its mortality rate is 18–35% in the pediatric population (6). However, the incidence and mortality rates of ARDS during the neonatal period remain unknown. Mid-term data from the ESPNIC/ESPR neonatal ARDS worldwide network concerning the epidemiology of neonatal ARDS revealed that 162 neonates recruited prospectively had a mortality rate of 17.3% at the 36th postmenstrual week (7).

Inflammation plays a key role in the pathophysiology of ARDS, as it triggers endothelial cell activation, capillary leakage, and protein-rich fluids in the alveolar space, as well as increasing the secretion of proinflammatory cytokines that can prompt surfactant inactivation. Surfactant deficiency causes reduced compliance, normal, or slightly increased resistance in diffuse lung tissue, and hypoxemic respiratory failure (2). Commonly seen neonatal respiratory disorders such as meconium aspiration, pneumonia, and hemorrhage affecting the alveolar–epithelial layer are classified as direct triggers for ARDS. Conditions corrupting the alveolar–capillary layer are classified as indirect triggers, including sepsis, asphyxia, and *in utero* inflammation. The data of the ESPNIC/ESPR neonatal ARDS worldwide network indicate that sepsis is the most common trigger (39.1%), followed by pneumonia (29.1%); meconium, blood, or milk aspiration (27.2%); asphyxia (12.6%); and pulmonary hemorrhage (11.9%) (7). The patients that we present here exhibited late-onset neonatal sepsis; sepsis was proven by blood culture in Case 2 and by clinical and laboratory findings in Case 1.

Treatment of ARDS centers on adequate ventilation, improved oxygenation, adequate perfusion, and the reduction of inflammation (8). To improve lung function and gas exchange, an open lung strategy combined with exogenous surfactant was found to be efficient in a neonatal ARDS model (9). Alongside ventilation strategies, therapeutic agents such as surfactants, inhaled nitric oxide, sildenafil, prostacyclin, and corticosteroids can be used to improve oxygenation and ventilation (8). Studies on exogenous surfactant treatment among pediatric ARDS patients showed improved oxygenation but no difference in ventilation support or mortality. Although the Pediatric Acute Lung Injury Consensus Conference Group (PALICC) does not recommend the routine use of surfactant in ARDS treatment, it was highlighted that specific patient populations may benefit from specific dosing and delivery regimens (10). Neonates may frequently develop surfactant inactivation and secondary dysfunction caused by direct or indirect triggers, especially in cases of pulmonary parenchymal diseases, and during this specific period exogenous surfactant treatment may be considered (11).

The anti-inflammatory effect of budesonide is clearly defined, and its combination with exogenous surfactants may advance its distribution and effect. In an animal study investigating the biophysical profile of a suspension combining surfactant and budesonide, the mixed suspension was observed to be biophysically and chemically stable (12). In an animal model of meconium aspiration syndrome, the combined therapy improved functional lung measurements, mean airway pressure, and oxygenation index and had a longer-lasting effect than either of the single administrations (13).

Budesonide combined with surfactant was also used in a clinical trial among preterm infants during the acute phase of respiratory distress syndrome in order to prevent the development of bronchopulmonary dysplasia (BPD) (4). In that study, the combination of 100 mg/kg beractant and 0.25 mg/kg budesonide was used endotracheally and, as a result, decreased pulmonary inflammatory status was achieved. The group receiving surfactant and budesonide had lower rates of death and BPD compared to the control group.

The definition of neonatal ARDS covers a group of respiratory conditions that mainly have similar pathophysiologies resulting in severe hypoxemic respiratory failure. Marked reduction of the alveoli, decreased alveolar surface area, and altered cardiopulmonary physiology in preterm infants with BPD leads to acute episodes of pulmonary decompensation characterized by hypoxemia (14). Unlike the recently defined neonatal ARDS criteria, BPD exacerbation does not have a strict definition. It is generally characterized by worsening of oxygenation, recurrent hypoxic episodes, and increased need for ventilatory support. In moderate or severe cases, pulmonary hypertension arising as a result of the abnormalities of the pulmonary vasculature and parenchymal lung disease may worsen the clinical picture (15). Distinct from the clinical exacerbations in cases of BPD, the definition of neonatal ARDS also requires acute onset (i.e., within a week), oxygenation index of >4, and lung imaging showing diffuse bilateral opacification with no cardiogenic origins for pulmonary edema (1). BPD has not been defined as an exclusion criterion for the diagnosis of neonatal ARDS. Before the definition of the Montreux criteria, some of the clinical pictures defined as BPD exacerbation would probably have fulfilled the criteria of neonatal ARDS. The specific contribution of BPD to the development of neonatal ARDS is not known. Disrupted pulmonary growth and immune function in preterm infants with BPD could potentially act as a basis for predisposal to neonatal ARDS. Until recently, neonatal ARDS was not defined well for newborns. Therefore, this may have led to the under-diagnosis of ARDS in this population with underlying BPD (16). Following the Montreux definition, cases diagnosed as neonatal ARDS on the basis of BPD will probably increase, and new cases may provide more accurate data in terms of management strategies. In both of our cases, we encountered acute worsening of pulmonary status, like in most preterm infants with BPD, but the severity of hypoxemia defined objectively with the oxygenation index and diffuse pulmonary parenchymal changes led us to consider a neonatal ARDS diagnosis on the basis of BPD. Whether a case is BPD exacerbation or neonatal ARDS, no specific therapy exists, and treatment of the underlying condition is essential. The most certain distinctions between the treatment of these conditions seem to be related to ventilation strategies and exogenous surfactant therapy. In pediatric patients, a ventilation strategy allowing lower tidal volumes and higher PEEP levels has been considered appropriate in ARDS management, similar to neonatal RDS management. In contrast to a low tidal volume and high PEEP strategy, larger tidal volumes delivered at slower ventilation rates with longer inspiratory times were recommended for BPD patients to improve the distribution of ventilation and minimize gas trapping (14, 17). Since the definition of ARDS is very new, specific ventilation strategies are not yet established for newborns. Because of the diffuse lung opacity pattern, we recruited the patients first and then switched to HFO mode to provide higher MAP levels and lower tidal volumes. Another difference in the treatments of ARDS and BPD exacerbation appears to be exogenous surfactant therapy. Unless there is evidence of secondary surfactant deficiency, surfactant treatment has not been recommended in cases of BPD exacerbation. Despite the fact that surfactant abnormalities in ARDS are not the essential pathogenic variables, surfactant insufficiency may result from primary or secondary inactivation of pulmonary surfactant in the alveolar cavity. Collapse and pulmonary edema worsen in the alveoli due to lack of surfactant, leading to the characteristic pathophysiology of ARDS. Some studies have demonstrated that exogenous surfactant may improve outcomes in infants and children (2). In our cases, because of the partial response to supportive management and ventilator management directed at ARDS, we considered exogenous surfactant treatment. In Case 1, the effect of a single dose of surfactant lasted only a few hours. Therefore, we decided to use budesonide combined with surfactant treatment to (i) minimize the inactivation of surfactant, (ii) utilize the local anti-inflammatory effects of budesonide, and (iii) improve the distribution of budesonide with the surfactant as a vehicle.

Neonatal ARDS is considered a novel diagnosis in the neonatology field. Our current scientific knowledge is largely based on ARDS studies in pediatric and adult populations. Establishing neonatal multicenter studies could have significant benefits for better understanding ARDS in the neonatal population in terms of etiological factors, clinical characteristics, and therapeutic options targeting better outcomes.

## Conclusion

Knowing the potential anti-inflammatory effects of budesonide on lung injury and that its instillation with surfactant promotes surfactant distribution and may protect against surfactant inactivation, we examined combined treatment with budesonide and surfactant in two cases, which we believe are the first cases of neonatal ARDS treated with endotracheal surfactant with budesonide to be reported.

Since ARDS is seldom recognized in newborns, we encourage clinicians to consider the diagnosis of neonatal ARDS and we emphasize that budesonide combined with surfactant may be a novel therapeutic option in treating neonatal ARDS.

## Data Availability Statement

All datasets generated for this study are included in the article/supplementary material.

## Ethics Statement

Written informed consent was obtained from the parents of the participants for the publication of this case report.

## Author Contributions

BD and FT contributed to the study's conception and design. BD and MC participated in obtaining the clinical data. BD and FT wrote the manuscript. ND and HO critically revised it. All authors have approved this final version of the manuscript and have agreed to be accountable for all aspects of the work.

## Conflict of Interest

The authors declare that the research was conducted in the absence of any commercial or financial relationships that could be construed as a potential conflict of interest.
